# Genetic diet interactions of *ACE*: the increased hypertension predisposition in the Latin American population

**DOI:** 10.3389/fnut.2023.1241017

**Published:** 2023-10-26

**Authors:** Ana Karina Zambrano, Santiago Cadena-Ullauri, Patricia Guevara-Ramírez, Viviana A. Ruiz-Pozo, Rafael Tamayo-Trujillo, Elius Paz-Cruz, Adriana Alexandra Ibarra-Rodríguez, Nieves Doménech

**Affiliations:** ^1^Centro de Investigación Genética y Genómica, Facultad de Ciencias de la Salud Eugenio Espejo, Universidad UTE, Quito, Ecuador; ^2^Grupo de investigación identificación genética-IdentiGEN, FCEN, Universidad de Antioquia, Medellin, Colombia; ^3^Instituto de Investigación Biomédica de A Coruña (INIBIC)-CIBERCV, Complexo Hospitalario Universitario de A Coruña (CHUAC), Sergas, Universidad da Coruña (UDC), La Coruña, Spain

**Keywords:** *ACE*, nutrigenetics, traditional diet, cardiovascular disease, genetic adaptation, polymorphism, Latin America

## Abstract

Hypertension is one of the primary risk factors associated with cardiovascular diseases (CVDs). It is a condition that affects people worldwide, and its prevalence is increasing due to several factors, such as lack of physical activity, population aging, and unhealthy diets. Notably, this increase has primarily occurred in low and middle-income countries (LMICs). In Latin America, approximately 40% of adults have been diagnosed with hypertension. Moreover, reports have shown that the Latin American genetic composition is highly diverse, and this genetic background can influence various biological processes, including disease predisposition and treatment effectiveness. Research has shown that Western dietary patterns, which include increased consumption of red meat, refined grains, sugar, and ultra-processed food, have spread across the globe, including Latin America, due to globalization processes. Furthermore, a higher than recommended sodium consumption, which has been associated with hypertension, has been identified across different regions, including Asia, Europe, America, Oceania, and Africa. In conclusion, hypertension is a multifactorial disease involving environmental and genetic factors. In Latin America, hypertension prevalence is increasing due to various factors, including age, the adoption of a “Westernized” diet, and potential genetic predisposition factors involving the *ACE* gene. Furthermore, identifying the genetic and molecular mechanisms of the disease, its association with diet, and how they interact is essential for the development of personalized treatments to increase its efficacy and reduce side effects.

## Introduction

Hypertension (high blood pressure) is one of the primary risk factors associated with cardiovascular diseases (CVDs). It is defined as blood pressure (BP) of ≥140/90 mmHg (1). The prevalence of hypertension is increasing worldwide due to several factors, including lack of physical activity, population aging, and unhealthy diets, especially those with high saturated fat and sugar intake and low in fruits, vegetables, and whole grains (2, 3).

Moreover, the increase in hypertension prevalence has primarily occurred in low and middle-income countries (LMICs), whereas high-income countries (HICs) experienced a decrease in hypertension prevalence (3, 4). In Latin America, approximately 40% of adults have been diagnosed with hypertension (5). Furthermore, the consumption of fast and processed foods has increased in the region, leading to a higher risk of chronic diseases such as diabetes, hypertension, and CVDs (3). A study by Defagó et al. (6) analyzed the dietary patterns in South America and their correlation with hypertension. The authors found that one of the predominant diets in the region contained a high intake of sweets, refined grains, processed meats, and snacks. In addition, they identified that this type of diet was positively associated with hypertension (6).

Hypertension is considered a polygenic disease with more than 150 genes associated with it (7). The renin-angiotensin system (RAS) (Supplementary Figure S1) is an associated factor that plays a central role in BP regulation by maintaining sodium and water homeostasis (8). Moreover, the RAS participates in intracrine, autocrine, paracrine, and endocrine signaling, suggesting it influences intra-and extracellular processes (9). The studies on the RAS and its genes aim to establish a relationship to the development of cardiovascular pathology like hypertension (10). Furthermore, RAS polymorphisms have been associated with protective and pathogenic effects on hypertension (9).

The angiotensin-converting enzyme gene (*ACE*), part of the RAS pathway, has been correlated with high BP. *ACE* gene encodes an enzyme that plays a crucial role in BP regulation and electrolyte balance. The primary function of the enzyme is to convert angiotensin I into angiotensin II, a vasoconstrictor and aldosterone–stimulating peptide that regulates blood pressure and fluid-electrolyte balance. This enzyme inactivates bradykinin, thereby increasing blood pressure. Genetic polymorphisms in the *ACE* gene strongly influence the serum level of *ACE* and blood pressure (11, 12). For instance, one common variant associated with the enzyme’s activity is the rs4343 (c.2328G > A), located in the 17 exon of the *ACE* that results in a synonymous variant (13). The variant has been related to increased susceptibility to migraine (13), hypertension due to a high saturated fat diet (14), *ACE* activity (15), salt-sensitive hypertension risk (8, 16), hypertension (17, 18), atherosclerosis (19), adiposity and blood pressure (20), among others.

Reports have shown that the Latin American genetic composition is highly heterogenic (21–23). Moreover, this genetic background is associated with several biological processes, including disease predisposition, treatment effectiveness, and how the people in the region respond to different dietary patterns, among others. For instance, Ogunniyi et al. (24) described high disparities in hypertension prevalence due to race and ethnicity. The authors mentioned that Hispanic and Black adults have an increased risk of developing hypertension, which is correlated with higher mortality and morbidity rates (24).

The present mini review aims to provide an overview of the complex relationships between diet, *ACE* polymorphisms, and hypertension, focusing on how these interactions affect diverse populations. By doing so, it aims to contribute to the understanding of hypertension and the implication for clinical practice and public health.

## Impact of dietary patterns on *ACE*

**Figure 1:**
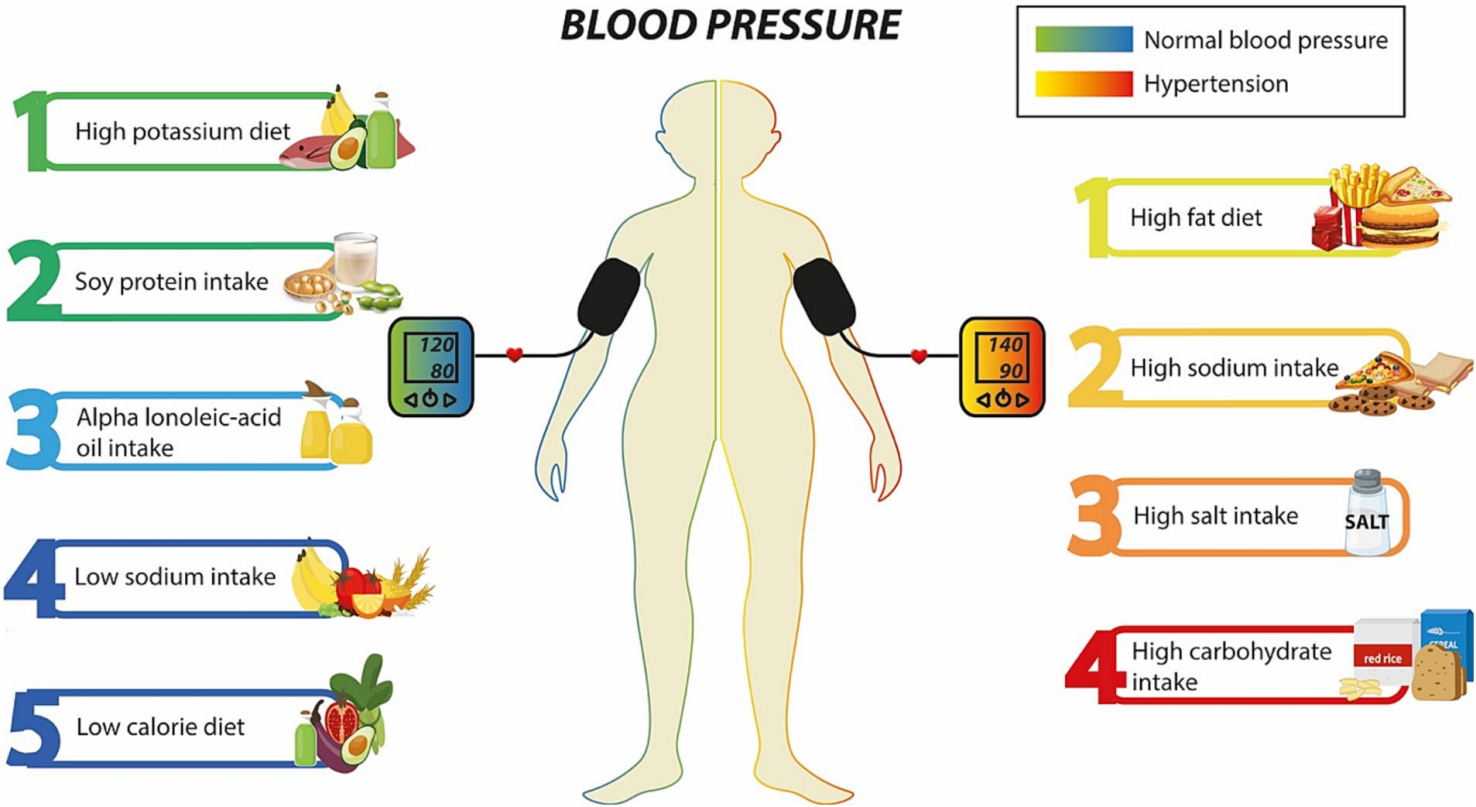
Dietary patterns associated with blood pressure. On the left side, the components correlated with a normal blood pressure are depicted; whereas on the right side, the factors related to hypertension are shown.

Recent studies have investigated the impact of dietary patterns on *ACE*, focusing on their potential to modulate *ACE* activity. This section aims to review the existing research on diet and its influence on *ACE*, with a particular emphasis on comparative studies that explore the effects of specific foods (Figure 1). For instance, Schüler et al. (14) studied forty-six Caucasian non-obese healthy twins with a median age of 31 ± 14 years. The researchers evaluated the effects of a high-saturated-fat (HF) diet under isocaloric conditions, compared to a diet rich in carbohydrates and low-fat, over a period of 6 weeks each. As a result, the authors found that the group that underwent a HF diet, had a 15% increase in circulating *ACE* concentrations and higher *ACE* expression in adipose tissue (14).

Furthermore, a study by Ogawa et al. (25) analyzed the impact of a 10% alpha-linolenic acid-rich flaxseed oil diet compared with high oleic safflower oil (control) on *ACE*. The research identified a significant decrease in *ACE* mRNA expression levels and *ACE* activity in the group that consumed the alpha-linolenic acid-rich diet compared to the control group (25). This study provides valuable information on the potential of alpha-linolenic acid-rich flaxseed oil as an *ACE* regulator, suggesting possible benefits in hypertension management.

Moreover, research by Tejpal et al. (26) described the association between *ACE* expression and activity with weight loss. The study included 32 participants from the University of Warsick, who were 18 years old or older, and not taking any medication. The mean BMI of the subjects was 28.4 ± 4.8 kg/m^2^ and 78% were females, and 22% males. The participants followed a 1,200 KCal calorie-restricted diet, and recorded physical activity, food intake, and urine collection. The authors identified that the *ACE* levels correlated with weight loss in patients with obesity and decreased during calorie restriction (26). Similarly, in a study by Harp et al. (27), the effects of dietary weight loss on *ACE* activity were analyzed. The project included 16 adults with obesity and a mean BMI of 35.7 ± 4.3 kg/m^2^. The researchers found that dietary weight loss decreased by 23% ± 12% *ACE* activity (27).

Emerging research suggests that dietary patterns and specific foods can modulate *ACE* gene expression and activity. Diets with an increased intake of potassium, soy protein, alpha-linolenic acid, and low in sodium have been shown to decrease *ACE* activity, potentially reducing the risk of hypertension (25, 28–30). Furthermore, similar studies have demonstrated the impact of diet on *ACE* function (27, 31). However, further investigation is required to elucidate the underlying mechanisms of this interaction and use this information to develop personalized dietary strategies that consider the *ACE* gene.

## Influence of the *ACE* polymorphisms on hypertension in response to the diet

As previously described, the *ACE* gene has been strongly associated with hypertension (7, 32). Moreover, single nucleotide polymorphisms (SNPs) within the gene have also been correlated with disease risk to varying degrees based on factors such as diet, individual traits, race, and region (7). Table 1 shows several risk alleles of *ACE* gene polymorphisms that have been associated with hypertension and the reported frequency of each SNP in Latin America, Europe, Asia, and Africa.

**Table 1:** Reported hypertension risk allele frequencies in Latin America, Europe, Asia, and Africa (33).

*ACE* polymorphism	Risk allele frequency in Latin America	Risk allele frequency in Europe	Risk allele frequency in Asia	Risk allele frequency in Africa
rs4290	C = 1	C = 1	C = 1	C = 0.89
rs4291	T = 0.36	T = 0.39	T = 0.33	T = 0.35
rs4305	A = 0.48	A = 0.45	A = 0.36	A = 0.81
rs4335	G = 1	G = 0.63	G = 0.78	G = 0.66
rs4343	A = 0.57	A = 0.46	A = 0.65	A = 0.74
rs4344	G = 0.53	G = 0.55	G = 0.36	G = 0.66
rs4353	A = 0.53	A = 0.54	A = 0.38	A = 0.64
rs4362	T = 0.48	T = 0.53	T = 0.38	T = 0.47
rs4363	G = 0.46	G = 0.53	G = 0.37	G = 0.43
rs1799752	NA	NA	NA	NA
rs7213516	A = 0.02	A = 0.001	A = 0	A = 0.16
rs7214530	G = 0.04	G = 0.001	G = 0	G = 0.21

Jeong et al. (16) conducted a Mendelian randomization study on a sample of 51,034 adults from Korea to investigate the association between sodium intake, hypertension, and genetic polymorphisms. The authors analyzed 1,282 alleles and found that the A allele of rs4343 increased the hypertension risk by more than 2.1-fold, and this risk was further amplified by high sodium intake (16).

Similarly, Wang et al. (34) analyzed 32 SNPs of the *ACE* gene in 1,024 hypertensive and 956 control participants. The authors reported that rs4343 was a risk factor for high pulse pressure levels associated with arterial elasticity and hypertension (34). Additionally, in the province where the study was performed, the diet included a high salt intake, and when the participants were overweight, the risk increased, suggesting a correlation between diet, obesity, and hypertension (34). These results align with those of Wang et al. (32), where a correlation between a high-salt diet and increased hypertension prevalence was described (32). More studies regarding the impact of rs4343 have been performed for different populations, including samples from Europe, and Asia, with similar outcomes, associating rs4343 with an increased hypertension risk (35). Furthermore, Schüler et al. found that the rs4343 *ACE* polymorphism was a biomarker correlated with higher *ACE* levels and a higher risk of hypertension (14). Similarly, in another study by the same group, the authors again observed increased *ACE* levels in response to a high-fat diet, which was associated with rs4343 and an increased risk of developing type 2 diabetes (36).

Furthermore, Martínez-Rodríguez et al. (37) analyzed the correlation between five *ACE* SNPs (rs4363, rs4362, rs4353, rs4344, rs4335, and rs4291) and essential hypertension in Mexican Mestizo individuals. The authors found that, under a dominant model, all the polymorphisms were associated with an increased hypertension risk. Moreover, by including the polymorphisms in haplotypes, one specific haplotype (*GGATG*) was related to a higher hypertension risk (37). Interestingly, the association remained significant even after considering factors such as smoking, age, gender, alcohol consumption, BMI, and triglycerides. Similarly, Ji et al. (38) described an association between rs4305 and hypertension in the Han Chinese population. Additionally, they found a correlation between *ACE* serum levels and BMI, triglycerides, and total cholesterol (38).

Likewise, Pachocka et al. (39) analyzed the correlation between *ACE*, environmental factors, and hypertension. The study included 73 adults (31 males and 42 females) with a BMI of >25 kg/m^2^. The authors described an association between rs1799752, hypertension, and carbohydrate intake. Individuals with the DD allele had a higher carbohydrate intake and an increased hypertension predisposition compared to those carrying the ID and II alleles. Moreover, they showed that people carrying the DD allele had an increased salt intake of more than 5 g/day, which may also be associated with a higher risk of hypertension (39). There are no reports of this SNP in the Latin American region.

Moreover, *ACE* variants in specific tissues has also been associated with cardiovascular phenotypes. For instance, Johnson et al. (40) evaluated *ACE* mRNA expression in heart tissues and genotyped the *ACE* locus. The study included the left-ventricle tissue from 65 heart transplant patients, including African American patients, at the Ohio State University. The authors found that three SNPs (rs7214530, rs4290, and rs7213516) affected *ACE* expression. Moreover, the SNPs rs4290 and rs7213516 were correlated with adverse cardiovascular outcomes, with an odds ratio of 6.16 for rs7213516 (40). In Latin America, the frequencies of rs7214530 and rs7213516 have been reported, whereas there are no reports of rs4290 (33).

In conclusion, *ACE* polymorphisms have been previously associated with an increased hypertension risk; hence, they could serve as biomarkers for hypertension predisposition. However, further studies are necessary to fully understand the interaction between genetic composition, hypertension, and diet.

## Discussion

Hypertension incidence is growing worldwide, and factors such as population aging, obesity, and an unhealthy diet, further increase the issue (7, 41). Furthermore, the genetic composition of a population could also significantly increase hypertension predisposition (32), highlighting the importance of gene–environment interactions involved in this disease. Moreover, LMICs are the most affected by the increase in hypertension prevalence, with more than 1.04 billion people living with this disease in these regions (42). Understanding the association between genetic factors and environmental influences is crucial for developing targeted disease management strategies.

Historically, the diet in Latin America has been primarily plant-based. For instance, the Maya culture consumed high quantities of corn, avocado, tomatoes, beans, and sweet potato. This diet was complemented by hunting, fishing, and turkey farming. Similarly, in the Inca civilization, their diet predominantly consisted of potatoes, which constituted a great source of carbohydrates, protein, and potassium. Meat consumption was rare, as cattle were mainly used for leather. Likewise, several other Latin American civilizations had similar plant-based diets (43). It is important to mention that these dietary patterns were prevalent before the colonization processes, which drastically changed the diet. However, based on genetic background analysis, most Latin American people still have a higher Native American ancestral proportion, which could still influence diet interactions and metabolism in the region (44).

Furthermore, Western dietary patterns, which include increased consumption of red meat, refined grains, sugar, and ultra-processed food, have spread across the globe, including Latin America, due to globalization processes (45, 46). For instance, according to the Pan American Health Organization (PAHO), the intake of high-saturated fats has increased in Latin America. The region has gone from consuming 53,458 kilotons of ultra-processed foods in 2000 to 79,108 kilotons in 2013, which may be correlated with increased hypertension prevalence (47).

Additionally, excessive salt consumption, which has been correlated with an increased hypertension risk, is also a common problem in the region. According to PAHO, the recommended daily salt intake is 5 grams, equivalent to 2 grams of sodium per day (48). However, studies have found that sodium intake is higher than recommended in the Latin American region. For example, in Brazil, sodium intake is 4.11 g/day; in Chile, it is 3.93 g/day; in Mexico, it is 3.1 g/day; and in El Salvador, it is 3.6 g/day (49–55).

Similarly, dysregulations in RAS have also been described as key factors correlated with hypertension pathogenesis (32). The RAS regulates blood pressure by modulating sodium concentration in plasma and can act locally or systematically through the action of the kidneys (9, 56). Notably, overactivity of the classical RAS pathway has been correlated with hypertension, while alternative pathways involving peptides, such as alamandine, and angiotensin, acting as antagonists of the classical RAS, have been associated with antihypertensive effects (57–59).

The most studied *ACE* polymorphism is rs4343, which, although a synonymous mutation resulting in the same amino acid (Thr776Thr), has been associated with a higher hypertension risk in both healthy and obese subjects (7, 16, 34, 36). Hypotheses suggest that the polymorphism could still influence gene expression by altering mRNA folding, leading to increased *ACE* protein synthesis (60, 61). In Latin America, databases report a risk allele frequency of 0.57 for rs4343 (Table 1) (33, 62), while, for the African population, the frequency is 0.74 (33, 62).

Table 1 presents several SNPs associated with hypertension in different regions. By analyzing the table, comparisons between populations can be made. For example, the frequency of risk alleles in Latin America is similar to the European population. However, Europe, comprised mostly of high-income countries (HIC), has lower hypertension rates compared to Latin America. In contrast, when comparing Latin America with Asia, the former carries more hypertension risk alleles, which aligns with hypertension rates in both regions. For instance, the Republic of Korea, and China are among the countries with the lowest hypertension prevalence for women, while Paraguay and the Dominican Republic are among the countries with the highest hypertension prevalence (3, 63).

On the other hand, the African population carries the highest number of hypertension risk alleles compared to Europe, Asia, and Latin America. Significantly, the World Health Organization (WHO) states that the African continent has the highest hypertension prevalence at 27% (64). Nevertheless, hypertension rates in Africa cannot be solely attributed to *ACE* SNPs, since hypertension is a multifactorial disease with a strong environmental component, including diet and physical activity. Thus, more research is needed to understand the genetic and environmental factors contributing to the condition.

Moreover, LMICs, including countries in Latin America, face another problem, which is limited healthcare access (3). According to PAHO, Latin America and the Caribbean are the most unequal regions in terms of health care access. For instance, only 7.7% of hypertension patients in LMICs have their BP under control (3). Furthermore, Horowitz et al. (65) conducted a study to analyze the perspectives of hypertension minority patients regarding diet modifications as part of their treatment. They found that patients have difficulties following the recommendations due to the costs, social situations, and withdrawal from their traditional diets, which could increase the risk and prevalence of hypertension (65).

In conclusion, hypertension is a multifactorial disease that comprises environmental and genetic factors. In Latin America, hypertension prevalence is increasing due to several factors, including aging, a “Westernized” diet, and possible genetic predisposition factors involving the *ACE* gene. Furthermore, understanding the genetic and molecular mechanisms of the disease, its association with diet, and how they interact is essential for the development of personalized treatments to increase its efficacy and reduce side effects.

## Future perspectives

Further research is required regarding the diet, hypertension, and genetic background. For instance, it is essential to continue with the genetic characterization of the Latin American populations and understand how they are associated with hypertension and other diseases. Moreover, functional analyses correlating the current Latin American diet with *ACE* activity should be conducted to elucidate the role of this diet on *ACE* activity.

## Author contributions

AZ and SC-U: conceived the idea, design, and writing. PG-R, VR-P, RT-T, EP-C, AI-R, and ND: written edition. All authors contributed to the article and approved the submitted version.
