# Supplementary figures and images for: Genetic diet interactions of ACE: the increased hypertension predisposition in the Latin American population
Source: Front Nutr. 2023 Oct 26;10:1241017. doi: 10.3389/fnut.2023.1241017 (PMC10640988; doi:10.3389/fnut.2023.1241017)

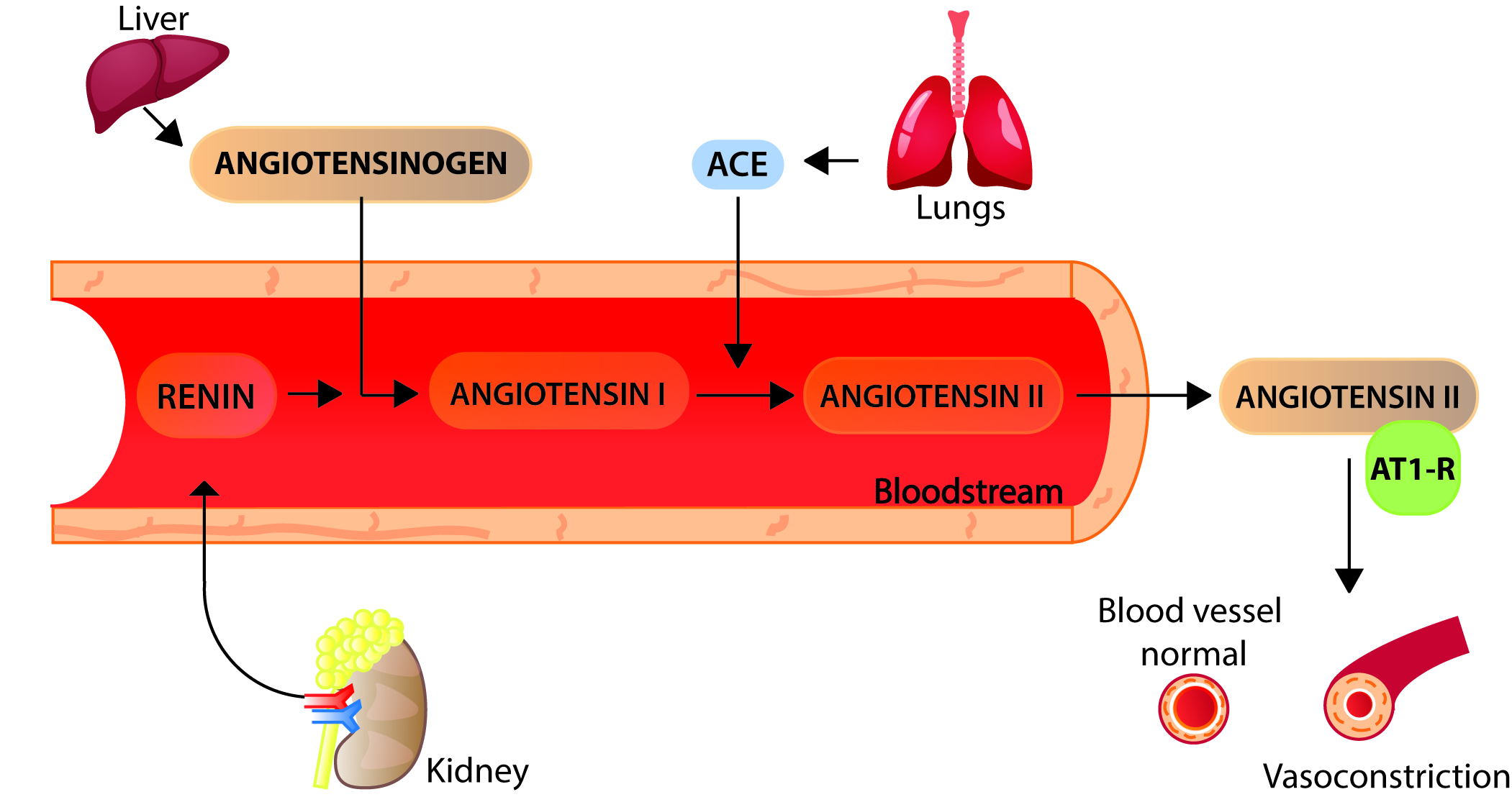

Supplement: Supplementary file 1 [file Image_1.JPEG]
